# Cholera

**Published:** 1849-02

**Authors:** 


### THE WESTERN JOURNAL.

THIRD SERIES.—VOL. III.—No. II.

LOUISVILLE, FEBRUARY 1, 1849.

### CHOLERA.

We have devoted much space in the present number of our Journal to this all-engrossing subject, but not more, we are persuaded, than will be acceptable to our readers. All things around us seem to indicate that the country is about to be visited once more by cholera in an epidemic form.

When the disease broke out in New Orleans, in December, the prevailing temperature was that of summer; on a change of the weather there was a marked abatement in the fatality of the cases, and the pestilence appears to be gradually passing away from that city. Although patients with cholera have been introduced into all the cities and towns on the Mississippi and its main tributaries, the epidemic, up to the present time, has not spread; but in most of these cities the imported cases have been accompanied by others of domestic origin, showing a disposition towards cholera which all professional men must have remarked. No increase of this tendency is yet manifest in Louisville, but rather a diminution, no case, since the first, having originated in the Marine Hospital, and no cases having been admitted for a fortnight past. The city may be considered healthy, notwithstanding that there is a predisposition to disorders of the bowels quite unusual at this season of the year. At the same time, there has not been anything remarkable in the weather. Rains have been frequent and copious, but not more so than we often have them in winter, and they have been succeeded by cold, bracing winds from the north-west, and frosts sufficiently protracted to enable the ice-men to fill their houses.

For a fortnight past the press at Cincinnati has been silent respecting cholera, and the alarm which began to be felt there in regard to it has subsided. At St. Louis it was not increasing on the 30th of January. Vicksburgh remained healthy up to the last of that month. At Lavaca ninety men of the 8th regiment of infantry, mustering 450 strong, are reported to have died of cholera up to the 25th of December.

The *origin* of cholera is a point which is still exciting much controversy, and, as will be seen by our extracts from foreign journals, the doctrine of its contagiousness, if not gaining advocates, is at least not losing ground abroad. The circumstances under which it has lately appeared in our country favor the idea that it is transmissible by persons, and that the sick may create an atmosphere around them in which nurses and others may contract the disease. It appears very manifestly to have been introduced into New York and New Orleans by ships from Havre; but the epidemic had no existence at the latter port when the packets sailed, nor has it yet been proved that any of the passengers, at least in the New York, were from points where it prevailed. And again, if the spread of the disorder in New Orleans seems to favor the idea of its contagiousness, its failure to spread in New York goes to show that the contagion is inoperative unless aided by favoring circumstances. In the former city, the Swanton arrived when the thermometer stood at 80 degrees, and the earth was drenched with incessant rains—in a condition of the atmosphere the most favorable to bowel complaints, and when sporadic cases of cholera were of frequent occurrence. In the latter, the state of things was entirely different. In one, the disease became epidemic; in the other it did not. The boats from New Orleans, while they have scattered cholera patients all over the Valley of the Mississippi, have nowhere propagated epidemic cholera.

